# Assessing the variability and predictability of adipokines (adiponectin, leptin, resistin and their ratios) in non-obese and obese women with anovulatory polycystic ovary syndrome

**DOI:** 10.1186/s13104-019-4546-z

**Published:** 2019-08-15

**Authors:** Christian Obirikorang, William K. B. A. Owiredu, Sandra Adu-Afram, Emmanuel Acheampong, Evans Adu Asamoah, Enoch Kwabena Antwi-Boasiakoh, Eddie-Williams Owiredu

**Affiliations:** 1 Department of Molecular Medicine, School of Medical Sciences, Kwame Nkrumah University of Science and Technology, Kumasi, Ghana; 2 School of Medical and Health Science, Edith Cowan University, Joondalup, Australia; 3 Department of Obstetrics and Gynaecology, Asafo-Boakye Specialist Hospital, Kumasi, Ghana

**Keywords:** Polycystic ovary syndrome, Anovulation, Adiponectin, Leptin, Resistin

## Abstract

**Objectives:**

To assess the variability and predictability of adiponectin, leptin, resistin and their ratios in non-obese and obese women with anovulatory polycystic ovary syndrome (aPCOS).

**Results:**

A total of 52 ovulatory controls (mean age = 31.63 ± 4.88 years, BMI = 25.33 ± 2.68 kg/m^2^); 54 non-obese (mean age = 32.11 ± 4.25 years, BMI = 25.72 ± 2.95 kg/m^2^) and 50 obese women with aPCOS (mean age = 33.64 ± 4.14 years, BMI = 39.19 ± 2.99 kg/m^2^) were recruited. The aPCOS group had lower adiponectin [13.0 (10.49–16.59) vs 18.42 (15.72–19.92) µg/ml, p < 0.0001], adiponectin: leptin ratio (A:L) [0.60 (0.35–0.88) vs 1.19 (0.92–1.37), p < 0.0001], and adiponectin: resistin ratio (A:R) [0.30 (0.21–0.43) vs 0.42 (0.32–0.62), p < 0.0001] but a higher leptin [20.02 (14.54–26.80) vs 16.17 (14.51–18.36) ng/ml, p < 0.0001] and leptin: resistin ratio (L:R) [0.53 (0.37–0.82) vs 0.40 (0.27–0.48), p < 0.0001] compared to the controls. The obese aPCOS group had lower adiponectin [11.04 (5.66–13.25) vs 14.18 (11.04–18.02), p < 0.0001 and 18.42 (15.72–19.92) µg/ml, p < 0.0001], A:L [0.36 (0.27–0.44) vs 0.78 (0.61–1.16), p < 0.0001 and 1.19 (0.92–1.37), p < 0.0001], and A:R [0.24 (0.17–0.38) vs 0.40 (0.23–0.58), p < 0.0001 and 0.42 (0.32–0.62), p < 0.0001] but a higher leptin [26.80 (14.28–32.09) vs 17.95 (14.86–21.26), p < 0.05 and 16.17 (14.51–18.36) ng/ml, p < 0.0001] and L:R [0.63 (0.46–1.03) vs 0.41 (0.30–0.61), p < 0.0001 and 0.40 (0.27–0.48), p < 0.0001] compared to the non-obese aPCOS and control group, respectively. A:L showed the best discriminatory power in predicting aPCOS (AUC = 0.83), followed by adiponectin alone (AUC = 0.79), L:R and leptin alone (both AUC = 0.69). Resistin alone had the poorest discriminatory power (AUC = 0.48).

**Electronic supplementary material:**

The online version of this article (10.1186/s13104-019-4546-z) contains supplementary material, which is available to authorized users.

## Introduction

Overweight and obesity are pervasive conditions which are considered global epidemic and threat to public health [1, 2]. Evidence suggest that obesity is associated with the risk of metabolic diseases such as diabetes, hypertension, cardiovascular disease, cancers and overall mortality [3–5]. There have also been reports of associations between obesity and infertility [6, 7], particularly among women due to the risk of anovulation [1, 8]. Anovulation is a common cause of infertility in women; responsible for 25–50% of female infertility [9, 10], of which polycystic ovary syndrome (PCOS) accounts over 90% of cases [11].

PCOS is a multisystem, endocrinological, reproductive and metabolic disorder characterized by oligo- and/or anovulation, hyperandrogenism, and polycystic ovaries [12]. Obesity-related adverse alterations in adipose tissue that predispose to metabolic dysregulation has been implicated in PCOS pathogenesis. These adverse alterations include derangements in bioactive cytokines and adipokines such as adiponectin, leptin, and resistin [13, 14].

Adiponectin is an adipokine secreted by adipose tissues, with anti-inflammatory, anti-atherogenic, cardio-protective, and insulin-sensitizing properties. Reduced adiponectin levels have been linked with obesity, T2DM, and PCOS [15–17]. Nonetheless, the relationship between altered adiponectin levels and PCOS remains debatable. While some studies report lower adiponectin levels in PCOS independent of BMI [13, 18], others report similar adiponectin levels in BMI-matched PCOS and controls [19, 20]. Leptin is an anorexigenic peptide hormone secreted by white adipose tissue [21]. Likewise, whereas some studies report a significant positive association between circulating leptin levels with high body fat independent of PCOS [22], others report no significant difference in circulating leptin levels between PCOS and age- and BMI-matched controls [23, 24] as well as between ovulatory and anovulatory women with PCOS [25]. Resistin is an adipocyte-derived polypeptide which have been associated with obesity, insulin resistance (IR) and cardiovascular risk [26, 27]. Similarly, some studies report comparable resistin levels between women with PCOS and controls [17, 27, 28] while others indicate that, irrespective of PCOS, there are elevated resistin levels in obese women compared to non-obese [29].

Thus far, reports regarding the alterations in adiponectin, leptin, and resistin in non-obese and obese women with PCOS remain inconclusive. Additionally, despite numerous previous studies highlighting the expediency of the ratios of these adipokines as a biomarker for obesity, IR, diabetes, coronary artery disease, and stroke [30–33], there is a dearth of information on their expediency as predictors of PCOS. This study aimed at assessing the variability and predictability of adiponectin, leptin, resistin and their ratios in non-obese and obese women with anovulatory PCOS.

## Main text

### Materials and methods

#### Study design/setting

This was a case–control study. Consecutive consenting women clinically diagnosed of aPCOS visiting the Obstetrics and Gynaecology units of Trust Care, Ruma and Asbury were included in the study as cases. PCOS diagnosis was based on the 2003 Rotterdam criteria [12]. All PCOS participants were anovulatory. Fertile (eumenorrheic) women visiting the hospital for routine check-up were included as controls. Relevant clinical data of each participant was extracted from the hospital’s archive. Women with Cushing syndrome, hyperprolactinemia, androgen-producing tumors, non-classic adrenal hyperplasia, active thyroid disease, and diabetes were excluded.

#### Study population and Anthropometric measurements

A total of 52 ovulatory, 54 non-obese and 50 obese women with aPCOS were included in this study. The weight was measured using a calibrated analogue scale (Seca, Hamburg, Deutschland). Height was measured using a stadiometer (Seca, Hamburg, Deutschland). Body mass index (BMI) was calculated by: BMI = weight/height^2^ (kg/m^2^) [34]. Obesity was defined according to the World Health Organization (WHO) criteria (BMI ≥ 30 kg/m^2^) [35]. Waist circumference (WC) and hip circumference (HC) were measured with a measuring tape; waist-to-height ratio (WHtR) = WC (m)/height (m), waist-to-hip ratio (WHR) = WC (m)/HC (m), body adiposity index (BAI) = (100 × HC (m))/(height (m) × √height (m)) − 18 [36] and visceral adiposity index (VAI) = (WC(m))/(36.58 + (1.89 × BMI)) × (TG/(0.81)) × ((1.52)/HDL-C) were calculated.

#### Blood sampling, processing and analysis

Five milliliters of venous blood was obtained from each participant and dispensed into gel separator tubes. The tubes were centrifuged at 1500×*g* for 10 min at 4 °C to obtain the serum which were stored at − 20 °C until analysis. Serum levels of adiponectin, leptin, and resistin were measured based on solid-phase sandwich Enzyme Linked Immunosorbent Assay (ELISA) technique (standardized with an intra- and inter-assay %CVs < 10%) (Green Stone Swiss Co Limited, China) according to the manufacturer’s instructions.

#### Statistical analysis

Statistical analysis was performed using the R Language for Statistical Computing version 3.6.0 [37]. Chi squared test was used to assess significance of association between the participant characteristics and fertility status. Distribution of adipokines were presented with density plots. Hierarchical clustering by Spearman’s correlation was used to assess relationship between adipokines (and their ratios) and obesity indices. Independent t-test and one-way ANOVA with Tukey test or Mann–Whitney U and Kruskal–Wallis with Dunn’s test were used to test for significance of difference between groups where applicable. The receiver operating characteristic (ROC) curve analysis was used to evaluate the performance of the adipokines (and their ratios) in predicting aPCOS. A p value < 0.05 was considered statistically significant.

### Results

A total of 52 ovulatory controls (mean age = 31.63 ± 4.88 years, BMI = 25.33 ± 2.68 kg/m^2^); 104 aPCOS patients comprising 54 non-obese [mean age = 32.11 ± 4.25 years, BMI = 25.72 ± 2.95 kg/m^2^] and 50 obese women [mean age = 33.64 ± 4.14 years, BMI = 39.19 ± 2.99 kg/m^2^] were included in this study. A higher proportion of the study participants had tertiary education, were employed and did not consume alcohol. There was no statistically significant association between fertility status and baseline characteristics (Table 1).

**Table 1:** Baseline characteristics of the study population

Variable	Ovulatory control (n = 52)	PCOS (n = 104)	p-value	Non-obese PCOS (n = 54)	Obese PCOS (n = 50)	p-value
Age (years)	31.63 ± 4.88	32.85 ± 4.25	0.112^‡^	32.11 ± 4.25	33.64 ± 4.14	0.062^†^
Educational level			0.312			0.219
None/basic	5 (21.7)	18 (78.3)		12 (52.2)	6 (26.1)	
Secondary	14 (41.2)	20 (58.8)		12 (35.3)	8 (23.5)	
Tertiary	33 (33.3)	66 (66.7)		30 (30.3)	36 (36.4)	
Occupation			0.197			0.093
Unemployed	9 (47.4)	10 (52.6)		8 (42.1)	2 (10.5)	
Employed	43 (31.4)	94 (68.6)		46 (33.6)	48 (35.0)	
Informal	10 (29.4)	24 (70.6)		10 (29.4)	14 (41.2)	
Formal	33 (32.0)	70 (68.0)		36 (35.0)	34 (33.0)	
Frequency of exercise			0.073			0.064
Rarely	17 (25.4)	50 (74.6)		22 (32.8)	28 (41.8)	
1/week	22 (34.9)	41 (65.1)		26 (41.3)	15 (23.8)	
> 1/week	13 (50.0)	13 (50.0)		6 (23.1)	7 (26.9)	
Alcohol consumption			0.235*			0.405*
No	36 (30.5)	82 (69.5)		42 (35.6)	40 (33.9)	
Yes	16 (42.1)	22 (57.9)		12 (31.6)	10 (26.3)	
Smoking status						
No	52 (33.3)	104 (66.7)		54 (34.6)	50 (32.1)	–

Unless otherwise indicated, Chi squared test was used to assess significance of association between the baseline characteristics and fertility status

* Fisher exact test for test of association

^‡^Significance of difference comparing Ovulatory control and PCOS group using Independent t-test

^†^Significance of difference comparing Ovulatory control, Non-obese PCOS, and Obese PCOS group using One-way ANOVA

**Fig. 1:**
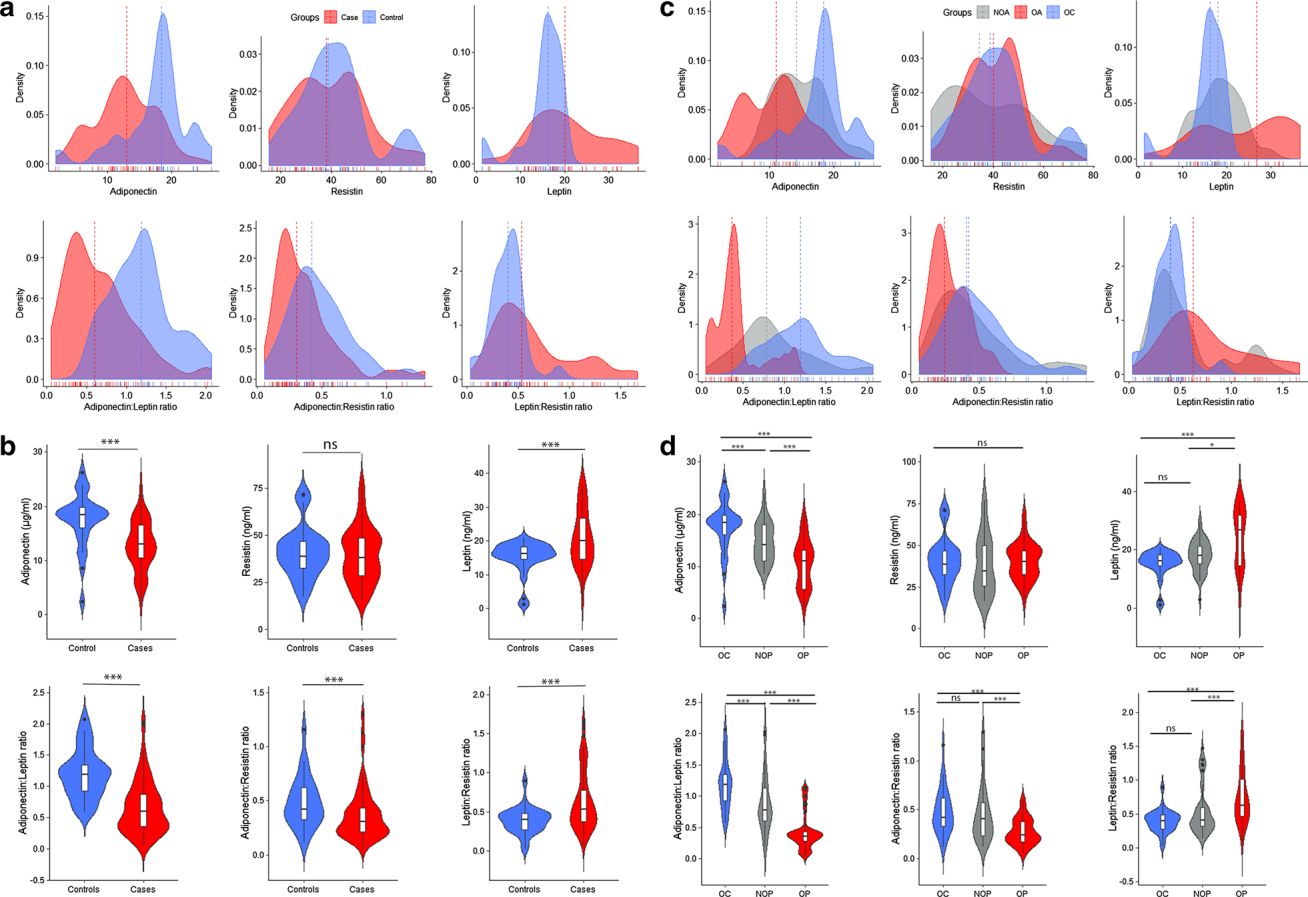
Variability of adipokines and their ratios between ovulatory and aPCOS groups. **a**, **c** Density plots showing distribution of adipokines. **b** Comparison of adipokines and their ratios between case and control groups. Mann–Whitney U test was used to assess significance of difference of adipokines and their ratios between ovulatory and aPCOS groups. **d** Comparison of adipokines and their ratios. Kruskal–Wallis H test was used to assess significance of difference of adipokines and their ratios between ovulatory (OC), non-obese (NOP), and obese PCOS (OP) groups. Post-hoc multiple comparisons was by Dunn’s test. *ns* not significant, *significant at p < 0.05, ***significant at p < 0.0001

The aPCOS group had a significantly lower adiponectin [13.0 (10.49–16.59) µg/ml vs 18.42 (15.72–19.92) µg/ml, p < 0.0001], adiponectin: leptin ratio (A:L) [0.60 (0.35–0.88) vs 1.19 (0.92–1.37), p < 0.0001], and adiponectin: resistin ratio (A:R) [0.30 (0.21–0.43) vs 0.42 (0.32–0.62), p < 0.0001] but higher leptin [20.02 (14.54–26.80) ng/ml vs 16.17 (14.51–18.36) ng/ml, p < 0.0001] and leptin: resistin ratio (L:R) [0.53 (0.37–0.82) vs 0.40 (0.27–0.48), p < 0.0001] compared to the controls (Fig. 1a, b).

The obese aPCOS group had a significantly lower adiponectin [11.04 (5.66–13.25) µg/ml vs 14.18 (11.04–18.02) µg/ml, p < 0.0001 and 18.42 (15.72–19.92) µg/ml, p < 0.0001], A:L [0.36 (0.27–0.44) vs 0.78 (0.61–1.16), p < 0.0001 and 1.19 (0.92–1.37), p < 0.0001], and A:R [0.24 (0.17–0.38) vs 0.40 (0.23–0.58), p < 0.0001 and 0.42 (0.32–0.62), p < 0.0001] but higher leptin [26.80 (14.28–32.09) ng/ml vs 17.95 (14.86–21.26) ng/ml, p < 0.05 and 16.17 (14.51–18.36) ng/ml, p < 0.0001] and L:R [0.63 (0.46–1.03) vs 0.41 (0.30–0.61), p < 0.0001 and 0.40 (0.27–0.48), p < 0.0001] compared to the non-obese aPCOS and control group, respectively (Fig. 1c, d).

**Fig. 2:**
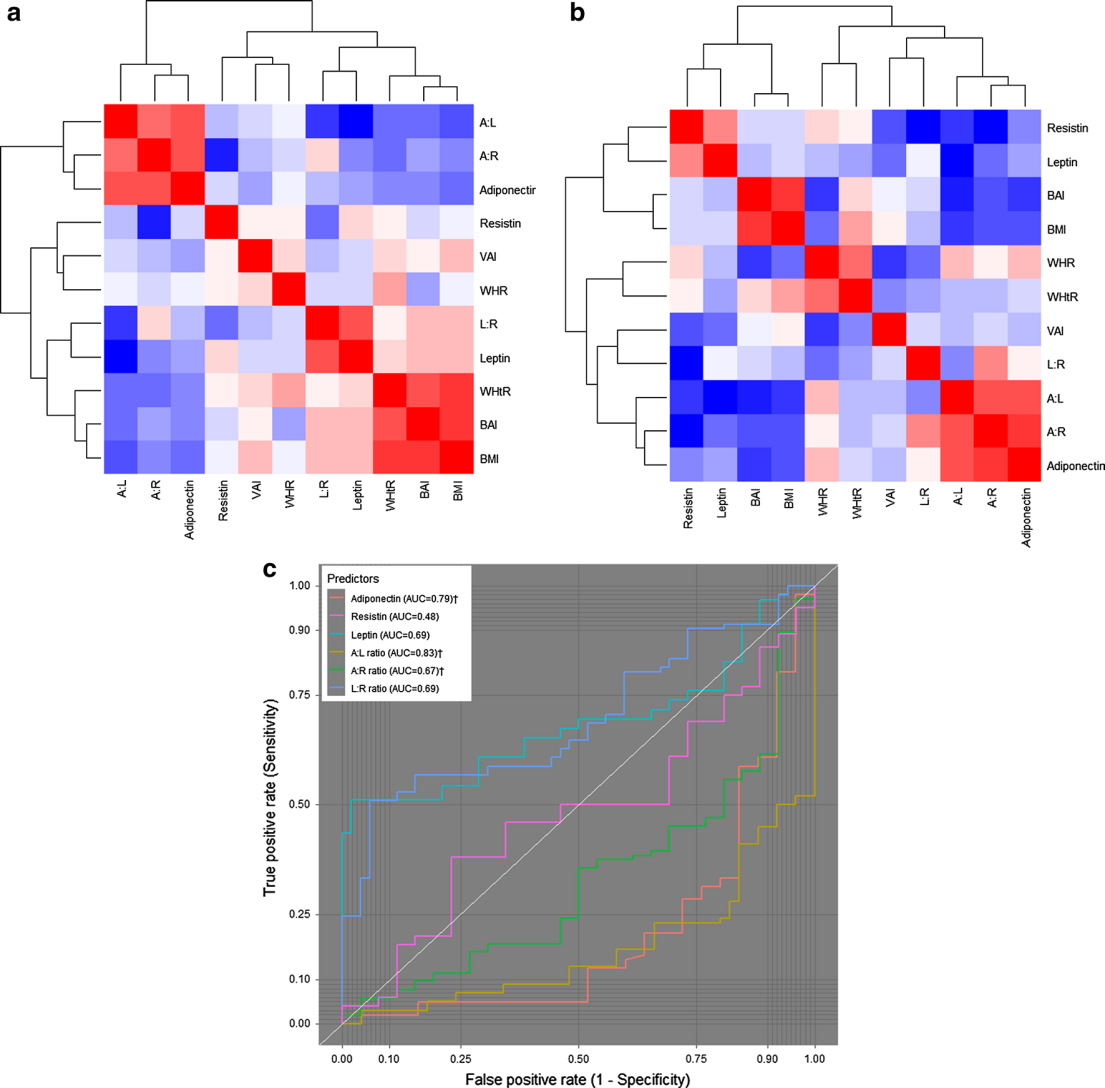
Correlational analysis and the performance of individual adipokines and their ratios in predicting aPCOS. **a** Correlation among aPCOS group. **b** Correlation among ovulatory control group. Hierarchical clustering by Spearman’s correlation was used to assess relationship between adipokines and their ratios with obesity indices. Blue-red coloration represents min (−) to max (+) correlation coefficient. **c** Receiver operating characteristic (ROC) curve was based on binary logistic regression and discriminant classification analysis for aPCOS and control groups. ^†^Test direction is negative (smaller test results indicate presence of condition). *A:L ratio* adiponectin:leptin ratio, *A:R ratio* adiponectin:resistin ratio, *L:R ratio* leptin:resistin ratio

Among the aPCOS group, adiponectin showed a significant negative correlation with BMI (rs = − 0.43, p < 0.0001), WHtR (rs = − 0.36, p < 0.0001), BAI (rs = − 0.35, p < 0.0001), and VAI (rs = − 0.19, p = 0.049). Leptin had a positive correlation with BMI (rs = 0.31, p = 0.001), WHtR (rs = 0.27, p = 0.007), and BAI (rs = 0.29, p = 0.003). There was no statistically significant correlation between resistin and obesity indices. A:L showed a negative correlation with BMI (rs = − 0.48, p < 0.0001), WHtR (rs = − 0.38, p < 0.0001), and BAI (rs = − 0.40, p < 0.0001). A:R showed similar correlations while L:R showed a positive correlation with BMI (rs = 0.27, p = 0.005) and BAI (rs = 0.29, p = 0.003) (Fig. 2a and Additional file 1: Table S1). Among the controls, adiponectin showed a significantly negative correlation with BAI (rs = − 0.33, p = 0.017) while resistin showed a positive correlation with WHR (rs = 0.31, p = 0.028) and WHtR (rs = 0.28, p = 0.044). There was no statistically significant correlation between leptin and obesity indices. A:L had a significant negative correlation with BMI (rs = − 0.36, p = 0.008) and BAI (rs = − 0.39, p = 0.004) while A:R showed a significant negative correlation with BMI (rs = − 0.29, p = 0.038) (Fig. 2b and Additional file 1: Table S2).

A:L presented with the best discriminatory power in predicting aPCOS (AUC = 0.83) followed by adiponectin alone (AUC = 0.79), and L:R and leptin alone (both AUC = 0.69). Resistin alone presented with the poorest discriminatory power (AUC = 0.48) (Fig. 2c).

### Discussion

Evidence suggest that, in PCOS, high body fat coupled with dysfunction of adipose tissue result in over-production of leptin, resistin and reduced expression of adiponectin. Levels of adiponectin have been shown to decrease in obesity and increase with weight loss [29]. It is considered a ‘beneficial’ adipokine in reproduction [38]. Leptin is constitutively secreted by adipocytes in proportion to the adipose mass [39]. In obesity, the levels of leptin are even more elevated due to leptin resistance [40]. Additionally, increased expression of the resistin gene has been observed in human pre-adipocytes, which decreased during adipocyte differentiation. Some studies report comparable adiponectin, leptin and resistin levels in PCOS [19, 24, 28] whiles others report lower adiponectin [18], higher leptin [22] and resistin levels [29] among women with PCOS than controls in relation to obesity. Thus, reports regarding the levels of adiponectin, leptin, and resistin in PCOS remain unresolved.

In this study, aPCOS patients had significantly lower levels of adiponectin, A:L, and A:R but higher leptin and L:R compared to the controls. Our finding is comparable to a study by Sarray et al. [28], who reported significantly lower levels of adiponectin, A:L, and A:R among women with PCOS compared to controls in Bahrain. A recent study by Baldani et al. also found significantly lower adiponectin and higher leptin among women with PCOS compared to controls in Croatia [41]. Upon stratification of aPCOS group by obesity status, we found the obese aPCOS group to have a significantly lower adiponectin, A:L, and A:R but higher leptin and L:R compared to the non-obese aPCOS and ovulatory group, respectively. This finding is in harmony with a study by Olszanecka-Glinianowicz et al. [17] who found serum adiponectin and A:R to be lowest in the obese PCOS subgroup compared to both the normal weight PCOS subgroup and the controls in Poland. In their study, serum resistin levels did not differ significantly between both non-obese and obese PCOS subgroups and the controls which is comparable to our study findings. Studies by Xiu et al. [42], Arikan et al. [43], and Seow et al. [44] also found similar serum resistin levels among controls, non-obese, obese women with PCOS. Furthermore, Sarray et al. [28] found markedly reduced A:L and A:R among obese women with PCOS compared to non-obese women with PCOS and controls. They also found lower L:R among obese women with PCOS though not statistically significant. Together with previous findings, our results corroborate the deposition that high body fat indeed paly pivotal roles in the pathogenesis of PCOS.

Also consistent with previous reports [39, 40, 45–47], we found that, with the exception of resistin, all other adipokines including their ratios were strongly and more correlated with various obesity indices among women with aPCOS compared to the controls. Specifically, adiponectin, A:L, and A:R showed a negative associations whereas leptin, and L:R correlated positively with the obesity indices. This finding is also coherent with studies by Sarray et al. [28] and Golbahar et al. [48].

In order to assess the predictive capabilities of the adipokines and their ratios, we employed the ROC curve analysis with reference to aPCOS. We found A:L to have the best discriminatory power in predicting aPCOS with an AUC of 0.83, followed by adiponectin alone (AUC = 0.79). Adiponectin and leptin are adipose tissue-derived hormones with contrasting relationship with the metabolic dysregulation [48]. Previous studies have highlighted A:L as a biomarker for obesity IR, and stroke [30–33]. Given the high AUC, our finding suggest that A:L could be a useful marker for aPCOS. This finding corroborates with a study by Golbahar et al. who found A:L to have a similarly high discriminatory power with comparable AUC of 0.86 among Bahraini women with PCOS [48]. Sarray et al. found a much higher discriminatory power (AUC of 0.94) for A:L in predicting PCOS in Bahrain [28]. The discrepancy in the predictive power may be attributed to differences in characteristics of the study population, sample size, and methods for biochemical analysis.

### Conclusion

This study shows significantly altered serum adiponectin and leptin levels but not resistin in Ghanaian women with aPCOS compared to healthy subjects. Obese aPCOS patients have the most altered levels of adipokines compared to non-obese aPCOS and healthy subjects. Adiponectin: leptin ratio is the best predictor of aPCOS compared to individual adipokines.

## Limitations

We did not assess androgens levels of the participants. Also, levels of insulin or IR was not evaluated. Furthermore, the relatively small sample size is a limitation of this present study. We recommend the use of larger sample size in future studies.

## Additional file

**Additional file 1.** Correlation co-efficient, lipid profile and anthropometric characteristics of the study population.

## Data Availability

The datasets supporting the conclusions of this article are included within the article and its additional file.

## Abbreviations

aPCOS: anovulatory polycystic ovary syndrome
BMI: body mass index
WHO: World Health Organization
T2DM: type 2 diabetes mellitus
WC: waist circumference
HC: hip circumference
WHR: waist to hip ratio
WHtR: waist to height ratio
BAI: body adiposity index
VAI: visceral adiposity index
ELISA: Enzyme Linked Immunosorbent Assay
