# Supplementary material for: Assessing the variability and predictability of adipokines (adiponectin, leptin, resistin and their ratios) in non-obese and obese women with anovulatory polycystic ovary syndrome
Source: BMC Res Notes. 2019 Aug 15;12:513. doi: 10.1186/s13104-019-4546-z (PMC6696689; doi:10.1186/s13104-019-4546-z)
Supplement: Supplementary file 1 — Additional file 1. Correlation co-efficient, lipid profile and anthropometric characteristics of the study population. [file 13104_2019_4546_MOESM1_ESM.docx]

**Table S1. Correlation co-efficient for PCOS group**

| Variables | Statistic | Adiponectin | Resistin | Leptin | A:L | A:R | L:R |
| --- | --- | --- | --- | --- | --- | --- | --- |
| BMI | rs | -0.433 | 0.057 | 0.309 | -0.476 | -0.348 | 0.272 |
|  | p-value | **<0.0001** | 0.563 | **0.001** | **<0.0001** | **<0.0001** | **0.005** |
| WHR | rs | 0.002 | 0.136 | -0.006 | -0.002 | -0.07 | -0.049 |
|  | p-value | 0.986 | 0.17 | 0.948 | 0.987 | 0.483 | 0.618 |
| WHtR | rs | -0.362 | 0.159 | 0.265 | -0.384 | -0.407 | 0.14 |
|  | p-value | **<0.0001** | 0.108 | **0.007** | **<0.0001** | **<0.0001** | 0.156 |
| BAI | rs | -0.353 | -0.02 | 0.29 | -0.401 | -0.251 | 0.286 |
|  | p-value | **<0.0001** | 0.837 | **0.003** | **<0.0001** | **0.01** | **0.003** |
| VAI | rs | -0.194 | 0.155 | -0.095 | -0.064 | -0.158 | -0.174 |
|  | p-value | **0.049** | 0.117 | 0.338 | 0.519 | 0.109 | 0.078 |

rs; Spearman correlation coefficient, p-values of significant variables in bold print

**Table S2. Correlation co-efficient for ovulatory control group**

| Variables | Statistic | Adiponectin | Resistin | Leptin | A:L | A:R | L:R |
| --- | --- | --- | --- | --- | --- | --- | --- |
| BMI | rs | -0.247 | 0.151 | 0.085 | -0.363 | -0.289 | 0.035 |
|  | p-value | 0.078 | 0.286 | 0.549 | **0.008** | **0.038** | 0.805 |
| WHR | rs | 0.157 | 0.305 | 0.013 | 0.186 | 0.275 | -0.174 |
|  | p-value | 0.101 | **0.028** | 0.928 | 0.105 | 0.149 | 0.217 |
| WHtR | rs | 0.102 | 0.281 | -0.029 | 0.018 | 0.021 | -0.064 |
|  | p-value | 0.474 | **0.044** | 0.837 | 0.898 | 0.883 | 0.650 |
| BAI | rs | -0.329 | 0.105 | 0.041 | -0.388 | -0.263 | 0.117 |
|  | p-value | **0.017** | 0.459 | 0.772 | **0.004** | 0.060 | 0.408 |
| VAI | rs | 0.022 | -0.305 | -0.157 | 0.044 | 0.120 | 0.126 |
|  | p-value | 0.880 | **0.028** | 0.267 | 0.756 | 0.397 | 0.373 |

rs; Spearman correlation coefficient, p-values of significant variables in bold print

**Table S3. Lipid profile anthropometric characteristics of the study population**

| Variable | Ovulatory Control (a) | PCOS | p-value‡ | Non-obese PCOS (b) | Obese PCOS (c) | p-value† | Significant pairs |
| --- | --- | --- | --- | --- | --- | --- | --- |
| TC (mmol/l) | 4.42±1.16 | 4.35±1.34 | 0.724 | 4.15±1.28 | 4.56±1.37 | 0.244 | - |
| HDL-C (mmol/l) | 1.46±0.35 | 1.17±0.47 | <0.0001 | 1.13±0.46 | 1.21±0.49 | <0.0001 | a&b, a&c |
| LDL-C (mmol/l) | 2.73±1.14 | 2.85±1.19 | 0.543 | 2.78±1.11 | 2.93±1.27 | 0.665 | - |
| TG (mmol/l) | 1.15±0.36 | 1.63±1.12 | 0.003 | 1.22±0.50 | 2.08±1.40 | <0.0001 | a&c, b&c |
| Weight (kg) | 62.31±9.16 | 77.25±14.23 | <0.0001 | 67.37±8.96 | 87.92±10.74 | <0.0001 | a&b, a&c, b&c |
| Height (m) | 1.57±0.08 | 1.59±0.14 | 0.230 | 1.62±0.08 | 1.56±0.18 | 0.039 | - |
| BMI (kg/m^2^) | 25.33±2.68 | 32.20±1.36 | 0.005 | 25.72±2.95 | 39.19±2.99 | <0.0001 | a&c, b&c |
| HC (m) | 0.98±0.08 | 1.11±0.12 | <0.0001 | 1.04±0.10 | 1.19±0.09 | <0.0001 | a&b, a&c, b&c |
| WC (m) | 0.89±0.08 | 0.95±0.12 | 0.002 | 0.89±0.10 | 1.01±0.10 | <0.0001 | a&c, b&c |
| WHR | 0.81±0.09 | 0.86±0.08 | 0.001 | 0.85±0.09 | 0.86±0.11 | 0.004 | a&b, a&c |
| WHtR | 0.57±0.05 | 0.60±0.09 | 0.020 | 0.55±0.06 | 0.65±0.09 | <0.0001 | a&c, b&c |
| BAI | 32.28±3.85 | 39.07±18.85 | 0.011 | 32.39±5.01 | 46.29±24.84 | <0.0001 | a&c, b&c |
| VAI | 1.60±0.50 | 3.21±3.19 | <0.0001 | 2.78±3.14 | 3.68±3.21 | <0.0001 | a&c, b&c |

‡; Significance of difference comparing Ovulatory control and PCOS group using Independent t-test

†; Significance of difference comparing Ovulatory control, Non-obese PCOS, and Obese PCOS group using One-way ANOVA
